# Knockdown of the lncRNA SNHG8 inhibits cell growth in Epstein-Barr virus-associated gastric carcinoma

**DOI:** 10.1186/s11658-018-0070-8

**Published:** 2018-04-27

**Authors:** Jing Liu, Chunxia Yang, Yufang Gu, Chong Li, Huamei Zhang, Wenfang Zhang, Xueqing Wang, Nan Wu, Chunyan Zheng

**Affiliations:** 1 Department of Nephology, Zibo, China; 2 Department of Gastrointestinal Surgery, Zibo, China; 3 Department of Medical Care, Zibo Central Hospital, 54 Gongqingtuan Rd, Zibo, Shandong 255036 People’s Republic of China

**Keywords:** SNHG8, Cell growth, shRNA, Epstein-Barr virus-associated gastric carcinoma

## Abstract

**Background:**

Epstein–Barr virus (EBV) infection is causatively associated with a variety of human cancers, including gastric cancer (GC), which has one of the highest mortality rates of all human cancers. Long non-coding RNAs (lncRNAs) show important regulatory roles in human GC. SNHG8 is a recently identified lncRNA that was reported to show abnormal expression pattern in GC. However, little is known of its biological function in EBV-associated GC.

**Methods:**

We used cell viability, colony formation and cell cycle assays to investigate the roles of lncRNA SNHG8 in the cell growth of EBV-associated GC.

**Results:**

The transcript levels of SNHG8 in the cultured EBV-associated GC cells were significantly higher in the cultured EBV-associated GC cells compared with the levels in normal human gastric mucosal cells and EBV-negative GC cells. Knockdown of SNHG8 with specific shRNAs inhibited cell proliferation and colony formation and arrested the cell cycle in the G0/G1 phase in vitro. We also found that knockdown of SNHG8 suppressed tumor growth in vivo.

**Conclusions:**

These data indicate the pro-oncogenic potential of SNHG8 in EBV-associated GC, meaning it is a latent therapeutic target for the treatment of this type of cancer.

## Background

Epstein-Barr virus (EBV) is a gamma herpes virus that infects over 90% of the world’s adult population. It can exist asymptomatically in the human system for a long time [1, 2].

A number of human malignancies are reported to be associated with EBV infection, including multiple types of Burkitt’s lymphoma, Hodgkin’s disease, nasal natural killer/T-cell lymphoma, nasopharyngeal carcinoma and gastric carcinoma (GC) [3–7]. EBV-associated GC constitutes almost a tenth of all GC cases, and about 75,000 new cases of EBV-associated GC occur worldwide each year [7, 8]. A recent study suggested that this 10% estimate might be too low, as 48/75 GC cases in the U.S. (64%) and 38/38 in Central America (100%) showed positive for EBV.

GC is the fourth most common cancer worldwide and ranks second on the cause list of cancer deaths [9]. EBV-associated GC is very difficult to treat: the complete elimination of tumor cells via surgical, radio-therapeutic and chemotherapeutic methods is challenging [10]. New therapeutic approaches are essential.

Long non-coding RNAs (lncRNAs) are currently defined as transcripts of ≥200 nt but without open reading frames (ORFs) [11]. Many studies have revealed that lncRNAs have regulatory functions, including modulation of apoptosis and invasion, reprogramming of induced pluripotent stem cells, markers of cell fate, and parental imprinting [12]. A link between altered expression of lncRNAs and cancer pathogenesis has been recognized, providing new insight into the genetic and molecular mechanisms of cancer [13–15]. In the case of gastric cancer, lncRNA dysregulation is associated with larger tumors, greater tumor invasion, more widespread metastasis, and lower survival rates [16, 17]. However, few studies have investigated lncRNAs in EBV-associated GC.

SNHG8, a novel small nucleolar guide RNA located on 4q26, was reported to have a high expression in EBV-associated GC [18]. Its precise biological role and mechanism of action in EBV-associated GC remain largely unclear.

Here, we explore the expression patterns of SNHG8 in EBV-associated GC and EBV-negative GC cell lines. We also examine the biological functions of SNHG8 in cell proliferation, cell cycle and apoptosis in vitro and in vivo.

## Methods

### Cell lines and culture conditions

Human gastric mucosal cell line GES-1 (Saierbio), EBV-associated GC cell lines GT38 and GT39 (American Type Culture Collection) and EBV-negative GC cell lines AGS and SGC7901 (Type Culture Collection of the Chinese Academy of Sciences) were used in this study. The cell lines were cultured in RPMI-1640 (Gibco; Thermo Fisher Scientific, Inc.) supplemented with 10% fetal bovine serum (FBS; ExCell Bio), 50 U/ml penicillin G and 50 U/ml streptomycin (Gibco) in at 37 °C in a 5% CO_2_ incubator. The medium was changed every 2 days and the cell line was passaged every 4 to 5 days.

### Cell transfection

Cells were grown in monolayers and conventionally passaged when the cell attachment rate reached 90%. The specific shRNA against SNHG8 was designed and synthesized at the Shanghai facility of Invitrogen. A negative control shRNA was synchronously synthesized. Cells were plated and cultured in growth media until the cell density reached 70%. Then shRNA transfection was conducted with Lipofectamine 2000 reagent (Invitrogen) based on the manufacturer’s protocol. Cells were harvested after 48 h.

### Short hairpin RNA-expressing plasmid construction, lentivirus packaging, cloning and stable transfection

To reduce the expression of SHNG8, human SHNG8 shRNA sequences were cloned into the pGIPZ-lentivirus vector (System Biosciences). Thereafter, SNHG8 knockdown vectors were constructed and sequenced. The empty pGIPZ vector without any insertion was used as a control. 293 T cells (Shanghai Research Institute of Chinese Academy of Sciences) were cultured in DMEM containing 10% FBS, maintained at 37 °C and transfected using Lipofectamine 2000 reagent with 3 μg pGIPZ-SNHG8-shRNAs, 6.0 μg PsPax2 and 3 μg pMD 2.G. The media were replaced with 10 ml fresh medium after incubation overnight. The virus-containing supernatants (pGIPZ-neg-shRNA-LV and pGIPZ-SNHG8-shRNA-LV) were collected at 48 h.

GT38 cells were infected and then selected using 4 μg/ml puromycin. The knockdown efficiency was measured using quantitative real-time PCR. Five days after infection, assays for cell proliferation, colony formation, cell cycle and cell apoptosis were performed.

In subsequent assays, the GT38 cells were divided into three groups in subsequent assays: blank control group (CON group; cells without infection), negative control (NC group; cells infected with pGIPZ-neg-shRNA-LV) and the SNHG8 knockdown group (KD group; cells infected with pGIPZ-SNHG8-shRNA-LV).

### Cell proliferation assay

Cell proliferation was assessed using the 3-(4, 5-dimethyl-2-thiazolyl)-2, 5-diphenyltetrazolium bromide (MTT) assay as previously described [19]. Briefly, after stable depletion of SNHG8, GT38 cells were seeded in 96-well plates at a density of 5 × 10^3^ cells/well and cultured for 1, 2 or 3 days. The supernatant was discarded, and 20 μl of MTT was added for another 4 h of incubation in RPMI-1640 supplemented with 10% FBS. Then, 150 μl of dimethylsulfoxide (DMSO) was mixed with the cells for 10 min. Absorbance of the cells in each well was observed at 570 nm under an Olympus absorption spectrophotometer for the cell number calculation. All the experiments were repeated three times.

### Clonogenic assay

The clonogenic assay was performed with a modification of a previously published method. In brief, after completion of NC or specific shRNA transfection, GT38 cells were plated into 6-well plates in triplicate and at a cell density of 100 cells/well. Then, the cells were grown in RPMI-1640 containing 10% FBS in an atmosphere of 5% CO_2_ and 95% humidity at 37 °C for 14 days. After that, the cells were fixed and stained with crystal violet, followed by air-drying. Finally, the colonies were manually counted under an Olympus IX83 microscope.

### Cell apoptosis and the cell cycle assay

After transfection with NC or specific shRNA, cells were cultured in RPMI-1640 containing 10% FBS for another 48 h. Then the cells were trypsinized and apoptosis was detected using an Annexin V-FITC Apoptosis Detection Kit (BD Biosciences). Then, cells were pelleted and washed with cold PBS and suspended in cold PBS. The cells were then treated with Annexin V–propidium iodide (PI) in the dark at room temperature according to the manufacturer’s instructions. The cells were kept on ice in the dark and analyzed via flow cytometry using a FACSCalibur (BD Biosciences). The data were analyzed using Cell Quest software.

For cell cycle analysis, GT38 cells transfected with NC or specific shRNA were harvested after 48 h. The cells were fixed with 70% ethanol at − 20 °C overnight and stained with PI (Sigma-Aldrich) in the presence of Ribonuclease A (Takara Biotechnology) for 30 min at room temperature. The cell cycle distribution was analyzed via flow cytometry using a FACSCalibur (BD Biosciences). All the experiments were repeated three times.

### Quantitative RT-PCR

Total RNA was isolated from cell lines using TRIzol Reagent (Invitrogen). Two micrograms of total RNA were reverse transcribed to obtain cDNA using Moloney Murine Leukemia Virus Reverse Transcriptase (M-MLVRT; Progema) according to the manufacturer’s instructions. Quantitative PCR was conducted with 1 μl of cDNA using SYBR Green Taq Mix (Takara) on a Bio-Rad Real-Time PCR System. Glyceraldehyde 3-phophate dehydrogenase (GAPDH) was used as the internal control for normalization. The primers for SNHG8 were synthesized by Invitrogen. Their sequences were: SNHG8 primers, forward: 5’-AAGTTTACAAGCATGCGCGG-3′; reverse: 5′- TCAAACTGACGGTTCTCGGG-3′; GAPDH primers, forward: 5′- CGCTCTCTGCTCCTCCTGTTC-3′; reverse: 5’-ATCCGTTGACTCCG ACCTTCAC-3′.

The thermal cycling conditions were: 95 °C for 5 min, followed by 40 cycles of 95 °C for 30 s, 60 °C for 30 s and 72 °C for 1 min. The final extension was 72 °C for 5 min. Experiments were repeated at least three times and the relative expression of SNHG12 was calculated using 2^–△△CT^ method.

### Animal experiments

Five-week old male BALB/c nude mice were purchased from the Chinese National Rodent Laboratory Animal Resources. Animals were housed in a specific pathogen-free room in accordance with the current regulations and standards. All animals were allowed to acclimatize to their new environment for one week prior to use. Mice were randomly divided into 2 groups (6 mice/group): those receiving GT38 cells with SNHG8 depletion (shRNA) and the control group, which received non-transfected GT38 cells. For each group of mice, 5 × 10^6^ cells/mouse were subcutaneously injected in the right side of the dorsal area.

Mice were then monitored for the growth of tumors. The tumor size was measured every 7 days for 28 days and estimated using the equation length × (width)^2^ × 0.5. After 30 days, the mice were killed, the tumors were removed, and the weight of each tumor was recorded. All animal experiments were performed according to the guidelines approved by the Chinese Association of Laboratory Animal Care.

### Statistical analysis

All data in this study are expressed as means ± SD, and differences between groups were determined using analysis of variance (ANOVA) with SPSS version 18.0. *p* < 0.05 was considered statistically significant.

## Results

### SNHG8 expression was upregulated in EBV-associated GC cells

**Fig. 1:**
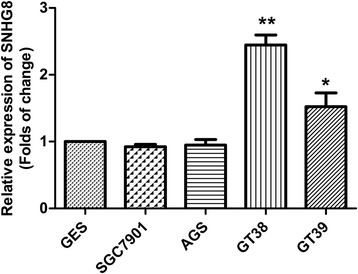
LncRNA SNHG8 expression was upregulated in EBV-associated GC cells. The relative SNHG8 levels are shown for normal human gastric mucosal cell line GES-1, EBV-negative GC cell lines SGC7901 and AGS, EBV-associated GC cell lines GT38 and GT39. *p < 0.01 and **p < 0.01 compared to GES-1.

To investigate the roles of the lncRNA SNHG8 in EBV-associated GC pathogenesis, the expression of SNHG8 in EBV-associated GC cell lines was assessed using qRT-PCR (Fig. 1). The results show that the relative SNHG8 mRNA expression in GT38 and GT39 cells was significantly higher than that in the normal human gastric mucosal cell line GES-1 and the EBV-negative GC cell line AGS and SGC7901.

### Knockdown of SNHG8 impaired proliferation and colony formation of GT38 cells in vitro

As shown in Fig. 1, the SNHG8 expression in GT38 was higher than that of GT39. Therefore, we selected GT38 cell line as the model to investigate the effect of SNHG8 on cell proliferation and apoptosis.

A specific shRNA against SNHG8 was used to stably deplete SNHG8 expression. Transfection of a negative control (NC) or specific shRNA was performed, and 90% of the cells expressed green fluorescence protein, indicating successful transfection efficiency (data not shown).

**Fig. 2:**
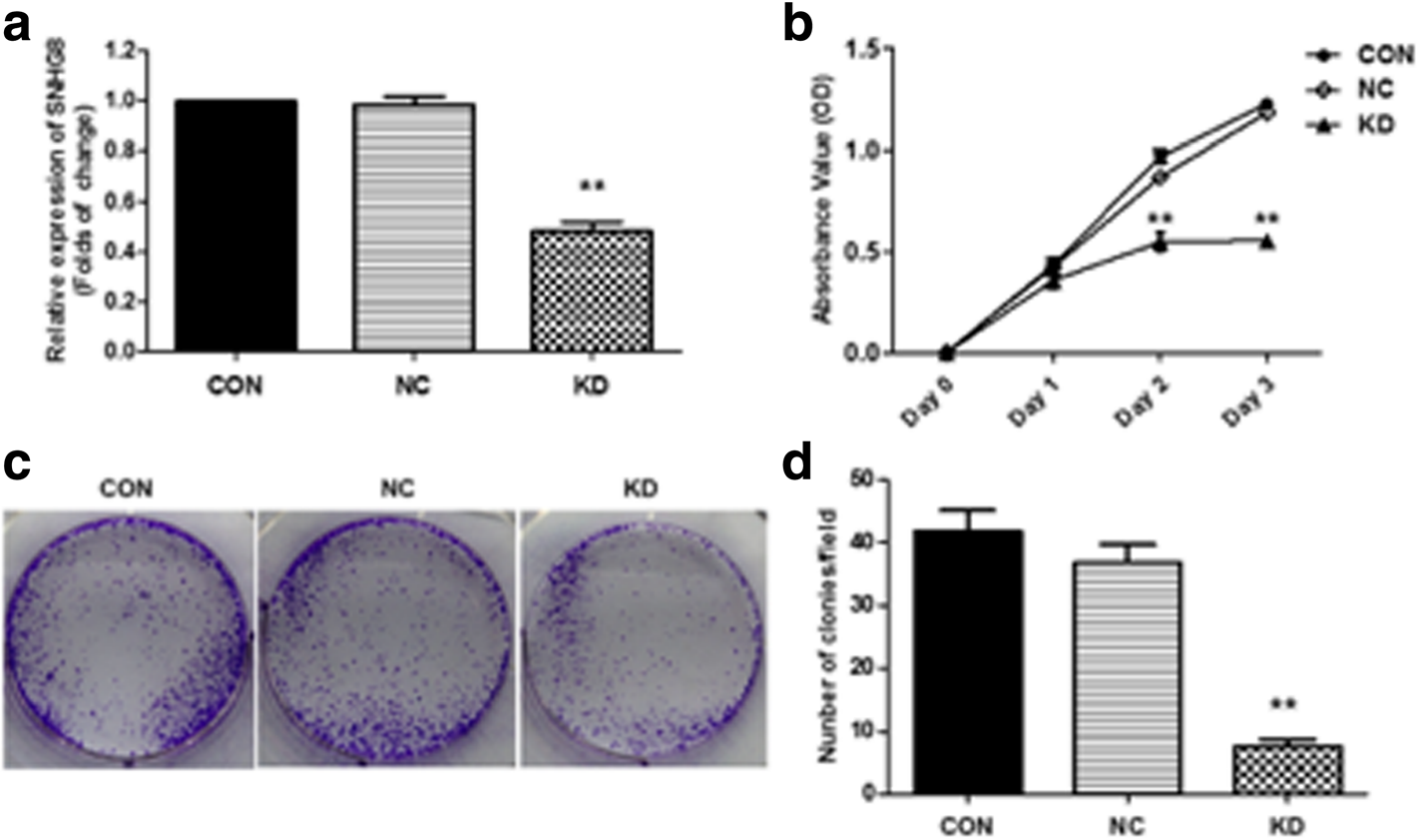
Knockdown of SNHG8 inhibits cell proliferation and clonogenic potential in GT38 cells. **a** The specific shRNA sh-SNHG8 decreased the SNHG8 level by up to 57% in GT38 cells. **p < 0.01. **b** The results of 3 consecutive days of cell viability assessments for GT38 cells with (KD group) or without (NC group) specific shRNA transfections and blank control cells (CON group). **c** GT38 cells with (KD group) or without (NC group) specific shRNA transfections and blank control cells (CON group) were subjected to colony formation assays. After crystal violet staining, colonies were visually observed. **d** Counting of colonies showed that colony numbers had significantly decreased in the KD group. **p < 0.01 compared with NC group. CON, blank control cells without transfection; NC, negative control; KD, SNHG8 knockdown

The specific shRNA sh-SNHG8 significantly decreased the SNHG8 level by up to 57% in GT38 cells (*p* < 0.01, Fig.2a). MTT assays showed that knockdown of SNHG8 obviously suppressed the proliferation rate of GT38 from day 2 (*p* < 0.01, Fig. 2b). Consistent with these results, the ability to form colonies by GT38 cells was also suppressed significantly after knockdown of SNHG8 (Fig. 2c). Counting of colonies showed that colony numbers significantly decreased compared with the NC and control groups (*p* < 0.01, Fig. 2d).

These results suggested that SNHG8 depletion has an inhibitory effect on the proliferation and clonogenic potential of EBV-associated GC cells.

### Knockdown of SNHG8 induced the apoptosis of GT38 cells

**Fig. 3:**
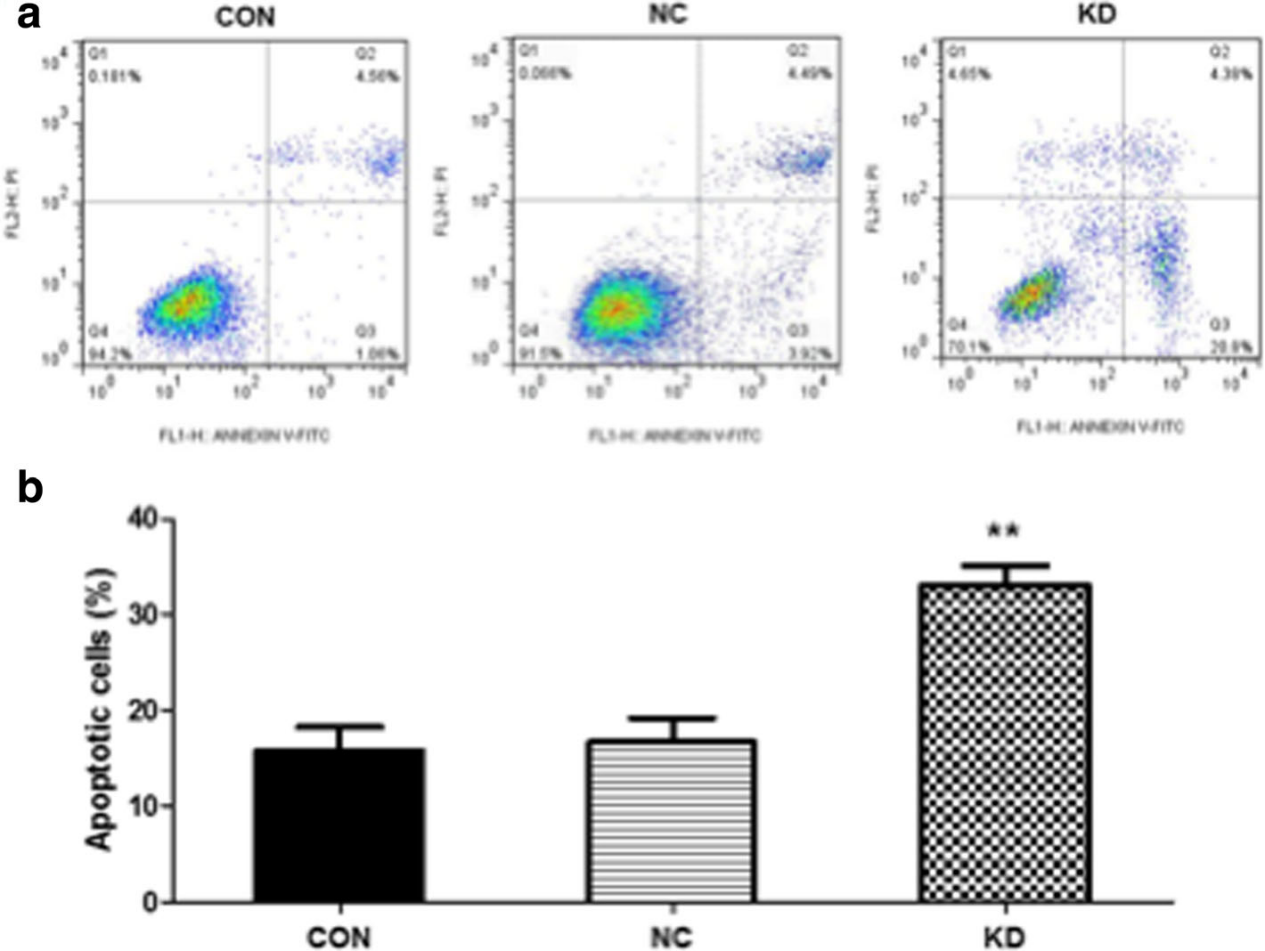
Knockdown of SNHG8 induced the apoptosis of GT38 cells. **a** Flow cytometric analysis for cell apoptosis in GT38 cells transfected with (KD group) or without the specific shRNA (NC group). **b** The corresponding statistical results. **p < 0.01 compared with NC group. CON, blank control cells without transfection; NC, negative control; KD, SNHG8 knockdown

To investigate the effect of SNHG8 on the cell apoptosis of EBV-associated GC cells, GT38 cells were stably transfected with the specific shRNAs (KD group) or without them (NC group), then stained with Annexin V and propidium iodide (PI), followed by detection using flow cytometry. As shown in Fig. 3, compared with the level for the NC group, the percentage of apoptosis in the KD group was significantly higher. These results indicate that knockdown of SNHG8 induced cell apoptosis of GT38 cells.

### GT38 cells were arrested in G0/G1 phase after knockdown of SNHG8

**Fig. 4:**
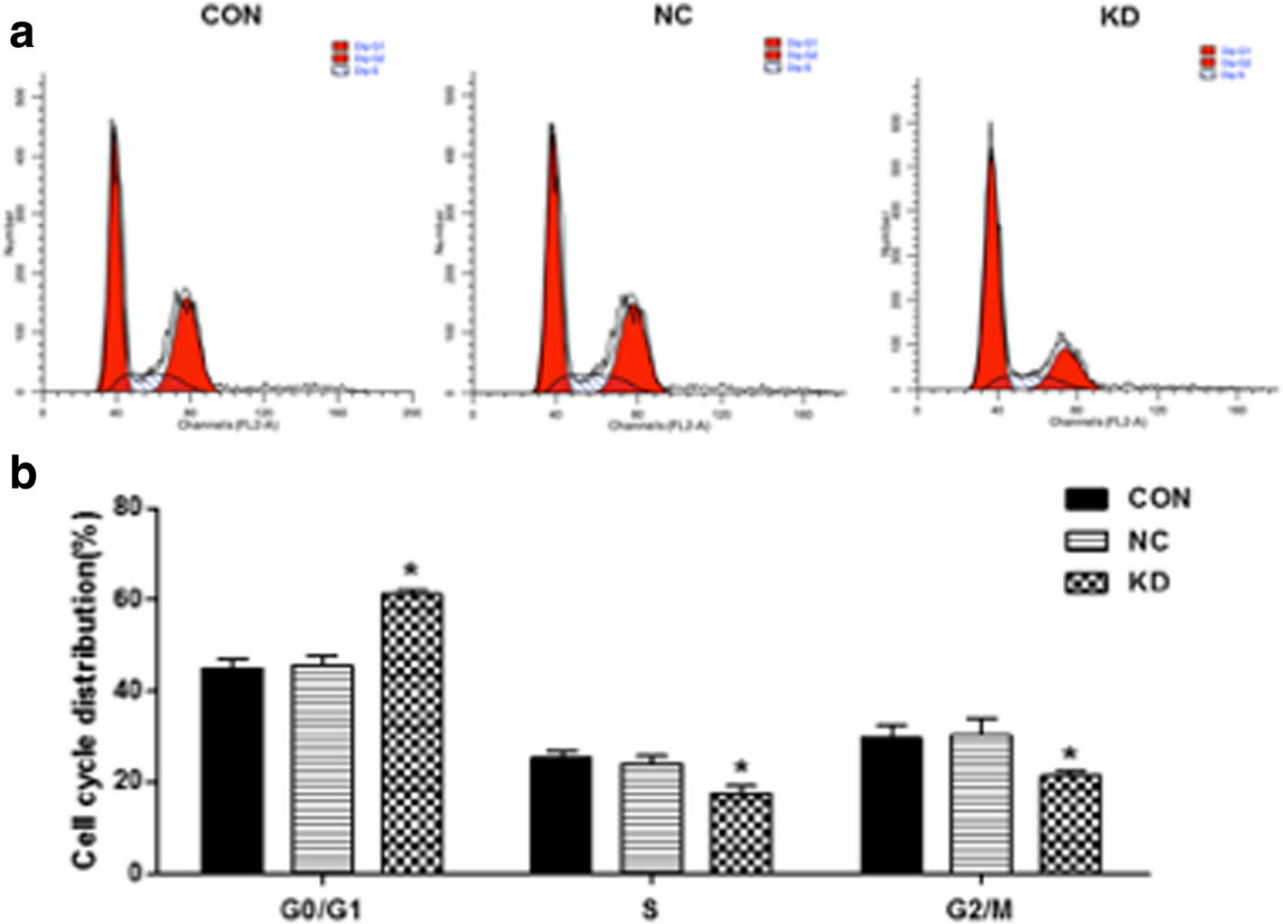
GT38 cells were arrested in the G0/G1 phase after knockdown of SNHG8. **a** Flow cytometric analysis for cell cycle progression in GT38 cells transfected with (KD group) or without (NC group) the specific shRNAs. **b** The corresponding statistical results. *p < 0.05 compared with NC group. CON, blank control cells without transfection; NC, negative control; KD, SNHG8 knockdown

Having found the effect of SNHG8 downregulation on the proliferation of the EBV-associated GC cell line GT38, we then examined the impact of decreased expression of SNHG8 on the cell cycle. Flow cytometric analysis showed a decrease in the percentage of cells in S phase and a marked accumulation of cells in G0/G1 phase in the KD group of GT38 cells compared with the NC group (Fig. 4). Compared with the control group, a greater proportion of SNHG8 knockdown cells remained in G0/G1 phase while fewer cells were in S phase. These results indicate that downregulation of SNHG8 expression in GT38 cells arrested in G0/G1 phase.

### SNHG8 knockdown inhibited tumor growth in vivo

**Fig. 5:**
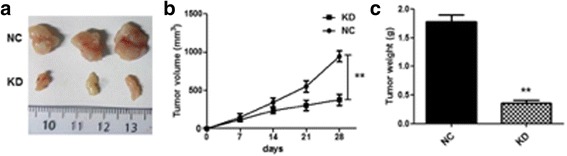
Effect of SNHG8 knockdown on EBV-associated GC growth in vivo. A subcutaneous xenograft tumor model of GT38 cells in nude mice was established. GT38 cells were transfected with sh-NC (CON group) or sh-SNHG8 (KD group; *n* = 6). **a** Tumors in mice inoculated with GT38 cells treated with sh-NC or sh-SNHG8. **b** Tumor size in the two groups. **c** Tumor weight in the two groups. **p < 0.01 compared with the CON group

To detect the effect of SNHG8 knockdown in vivo, a nude mouse xenograft model of EBV-associated GC cells was established. GT38 cells were stably transfected with sh-NC or sh-SNHG8 and subcutaneously injected into the nude mice (*n* = 6 for each group). As shown in Fig. 5a and b, SNHG8 knockdown inhibited tumor growth significantly after 28 days. In addition, the tumor weight on day 28 in mice inoculated with GT38 cells transfected with sh-SNHG8 was significantly lower than that of the controls (transfected with sh-NC; Fig. 5c). These in vivo results indicate that SNHG8 knockdown inhibited tumor growth.

## Discussion

Gastric cancer (GC) is a complex and highly heterogeneous disease [20]. Epstein-Barr virus (EBV) is absent in non-cancerous mucosa but present in all cancer cells, and has a clonal nature in neoplastic cells; therefore, it is considered to have a causal role in gastric carcinoma [8, 21]. EBV-associated GC constitutes almost 10–18% of all GC cases. Due to the challenges in treating this cancer, identifying additional targets that could serve as biomarkers for early diagnosis and treatment is urgent.

LncRNAs are a large subgroup of non-coding transcripts that have emerged as crucial regulators and prognostic markers in multiple cancers, including GC. Researchers who are committed to the search for effective gastric cancer diagnostic markers have found that gastric cancer is associated with the dysregulation of specific lncRNAs. For example, overexpression of the lncRNA H19 promoted proliferation, migration, invasion and metastasis of GC [17]. Another study showed that SNHG5 was significantly downregulated and associated with the TNM stage in patients with GC [22].

Our study investigated the role of a newly identified lncRNA, SNHG8, in cell growth, cycle and apoptosis in EBV-associated GC using the GT-38 cell line. We found that SNHG8 expression was significantly upregulated in EBV-associated GC cell lines, SNHG8 depletion by specific shRNA slowed down proliferative rates in EBV-associated GC cells and clonogenic potential was consistently impaired after SNHG8 knockdown. We transfected the same SHNG8 shRNA into GT-39 cells, another EBV-associated GC cell, and found the same effect of SNHG8 in GT-39 cells (data not shown), proving that SNHG8 has the same importance in different EBV-associated GC cells.

A xenograft model of EBV-associated GC in nude mice further confirmed that SNHG8 knockdown inhibited tumor growth in vivo. All these data suggest a tumorigenic role of SNHG8 in EBV-associated GC. Furthermore, GT38 cells that were depleted of SNHG8 exhibited increased cell apoptosis. Cell cycle was arrested at G0/G1 stage when SNHG8 was silenced.

## Conclusions

In summary, the findings from our study demonstrated that the downregulation of SNHG8 expression inhibits cell growth, arrests cell cycle and facilitates apoptosis. To the best of our knowledge, this study is the first to reveal a functional role for SNHG8 in EBV-associated GC. Using specific shRNA or developing novel targets against SNHG8 may be promising strategies for EBV-associated GC intervention or treatment.

## Abbreviations

DMSO: Dimethylsulfoxide
EBV: Epstein-Barr virus
FBS: Fetal bovine serum
GAPDH: Glyceraldehyde 3-phophate dehydrogenase
GC: Gastric cancer
lncRNAs: Long non-coding RNAs
MTT: 3-(4, 5-dimethyl-2-thiazolyl)-2, 5-diphenyltetrazolium bromide
NPC: Nasopharyngeal carcinoma
ORFs: Open reading frames
PI: Propidium iodide
shRNA: Short hairpin RNA
